# Molecular mechanism of substrate selectivity of the arginine-agmatine Antiporter AdiC

**DOI:** 10.1038/s41598-018-33963-1

**Published:** 2018-10-23

**Authors:** Eva-Maria Krammer, Andrew Gibbons, Goedele Roos, Martine Prévost

**Affiliations:** 1 Structure et Fonction des Membranes Biologiques, Université Libre de Bruxelles (ULB), Brussels, Belgium; 2 UGSF-Unité de glycobiologie structurale et fonctionnelle, University Lille, CNRS, UMR 8576, Lille, France

## Abstract

The arginine-agmatine antiporter (AdiC) is a component of an acid resistance system developed by enteric bacteria to resist gastric acidity. In order to avoid neutral proton antiport, the monovalent form of arginine, about as abundant as its divalent form under acidic conditions, should be selectively bound by AdiC for transport into the cytosol. In this study, we shed light on the mechanism through which AdiC distinguishes Arg^+^ from Arg^2+^ of arginine by investigating the binding of both forms in addition to that of divalent agmatine, using a combination of molecular dynamics simulations with molecular and quantum mechanics calculations. We show that AdiC indeed preferentially binds Arg^+^. The weaker binding of divalent compounds results mostly from their greater tendency to remain hydrated than Arg^+^. Our data suggests that the binding of Arg^+^ promotes the deprotonation of Glu208, a gating residue, which in turn reinforces its interactions with AdiC, leading to longer residence times of Arg^+^ in the binding site. Although the total electric charge of the ligand appears to be the determinant factor in the discrimination process, two local interactions formed with Trp293, another gating residue of the binding site, also contribute to the selection mechanism: a cation-π interaction with the guanidinium group of Arg^+^ and an anion-π interaction involving Glu208.

## Introduction

Both pathogenic and probiotic bacteria have developed various molecular strategies for resisting exposure to gastric pH (1.5–3.5) during their passage through the stomach, as they must survive this passage in order to colonize the gut^1,2^. One of these acid resistance (AR) mechanisms relies on the consumption of intracellular protons through the decarboxylation of an amino acid by a cytosolic enzyme. This enzyme is coupled with a membrane antiporter exchanging the decarboxylated product for the external amino acid^3^. Several distinct amino-acid-dependent AR systems have been characterized so far in *Escherichia coli*^4–8^, one of which being the arginine-dependent AR system^1,9,10^. This system involves arginine import into the cytosol by the arginine-agmatine antiporter AdiC followed by its decarboxylation to agmatine (Agm^2+^) by a cytosolic enzyme. The produced Agm^2+^ is then exported to the extracellular medium by AdiC. Since the pKa of the arginine carboxylate group is close to the typical gastric pH, two protonated forms of arginine, Arg^+^ and Arg^2+^, coexist in the stomach in roughly similar concentrations. The arginine-dependent AR system is only effective if Arg^+^ is transported by AdiC since the import of the additional proton bound to the divalent substrate counterbalances the proton-consuming decarboxylation of arginine by the cytosolic enzyme, leading to a net 0 proton exchange. An experimental study has recently shown that the arginine/agmatine exchange by AdiC is electrogenic at a pH typical of gastric conditions meaning proton consumption occurs during its transport cycle^11^. Additionally, in order for the AR system to function effectively, AdiC should also not import the freshly exported Agm^2+^ back into the cell. AdiC should therefore preferentially bind Arg^+^ from both Arg^2+^ and Agm^2+^ in the periplasmic medium.

The process through which AdiC distinguishes Arg^+^ from Arg^2+^ remains elusive to date. Two potential mechanisms have recently been advanced^11^: (1) a short-range mechanism based on the presence of a hydrogen bond interaction between the α-carboxylate group of the Arg ligand and the side chain of the binding site residue Ser26 observed in different arginine-bound AdiC crystal structures^12,13^ and (2) a mechanism relying on the global charge of the substrate. The short-range mechanism has been ruled out, since AdiC S26A displays similar arginine transport activity to the wild-type^11^. Another argument against the local hydrogen bonding mechanism is that AdiC preferentially transports the protonated form of citrulline (a transported arginine analog with an uncharged isosteric side chain) in comparison to its deprotonated form. These observations point towards a charge-based mechanism^11^. Such a mechanism has also been proposed to rationalize the transport selectivity of the analogous GadC, the antiporter component of the glutamate-dependent acid resistance system of *E*. *coli*^14^.

**Figure 1:**
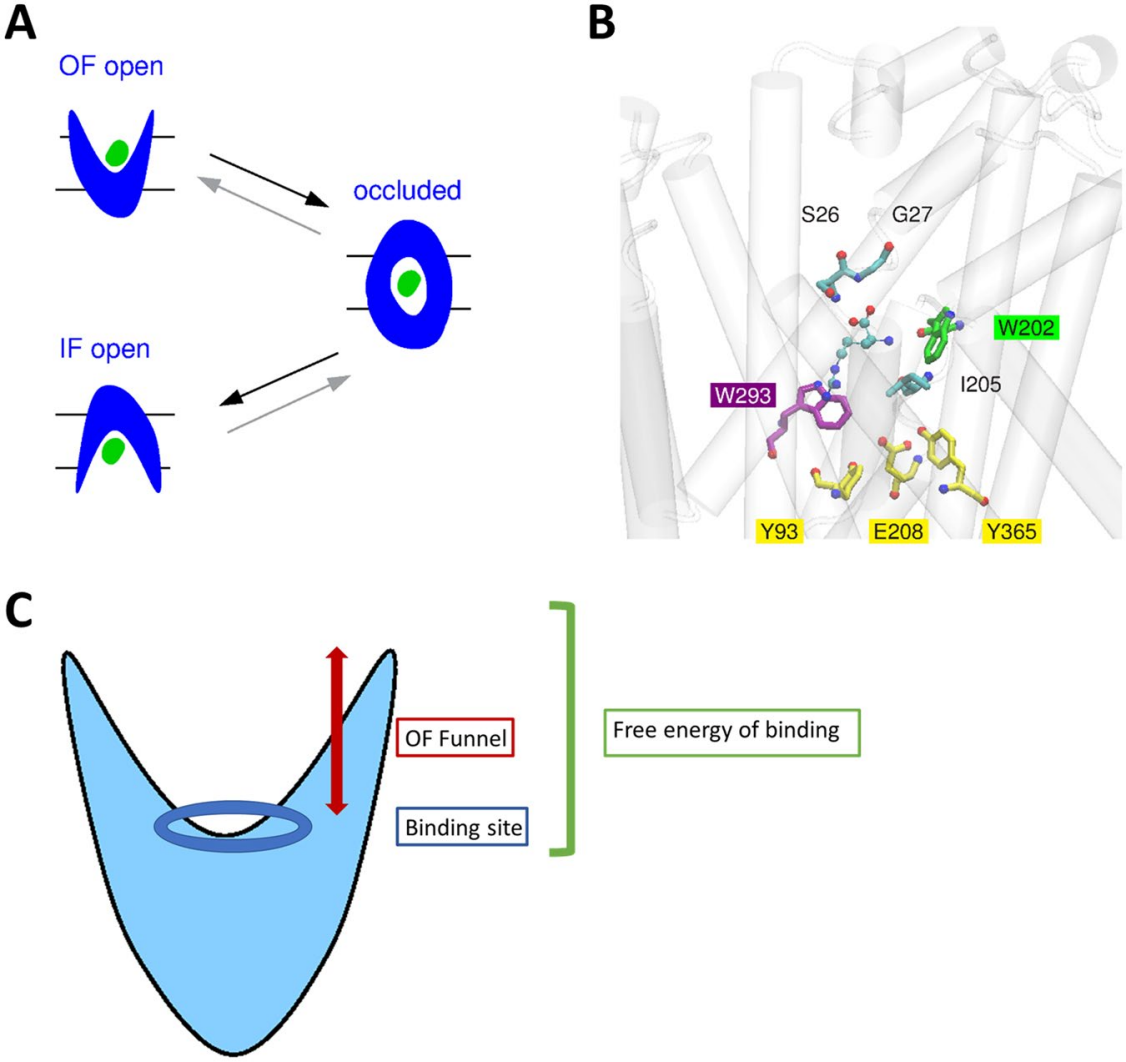
The AdiC transporter. (**A**) Schematic representation of the alternating access mechanism and of the main protein states occurring during transport. (**B**) Orientations of the gating residues in the OF open structure: the proximal and middle gate residues Trp202 and Trp293, respectively, and the distal gate residues Tyr93, Glu208, and Tyr365. The carbon atoms of the proximal, middle, and distal gate residues are respectively colored in green, purple, and yellow. The protein is shown as a transparent cartoon. The arginine ligand as well as Ser26 and Gly27 in the binding site are depicted as colored-atom sticks. (**C**) Schematic representation of the AdiC antiporter in the OF open conformation and possible selectivity filters for ligands: selection might occur during migration along the OF funnel to the binding site (red arrow) and/or in the binding site (blue ellipsoid), and/or it might result from the free energy balance of each ligand between the aqueous phase and the protein binding pocket (green bracket).

AdiC belongs to the amino acid-polyamine-organocation (APC) superfamily of secondary transporters, which all share a common fold^15^. Like many of these transporters, AdiC operates via the alternating access mechanism (Fig. 1A) during which the protein undergoes a transition between an outward-facing (OF) open and an inward-facing (IF) open conformation and opens or closes specific gating residues to keep the substrate exposed to one side of the membrane at a time. Analysis of the different AdiC structures has revealed three potential gates: the proximal and middle gating residues Trp202 and Trp293 and the distal gate formed by the interactions between Tyr93, Tyr365 and the acidic residue Glu208^13^ (Fig. 1B).

Theoretical calculations and simulations are a powerful means of understanding the molecular mechanisms of binding and transport of different substrates^16–18^. Several molecular dynamics (MD) and modeling studies have been performed to investigate AdiC substrate binding and its motions along its transport route^19–23^. Among them, one study has highlighted the impact of the protonated uncharged form of the gating residue Glu208 (Glu208^0^) on the release of Agm^2+^, but not Arg^+^, from the occluded binding pocket towards the periplasm^21^. To the best of our knowledge, however, none have yet addressed the issue of the discrimination of the two different protonation states of arginine by AdiC upon binding from the periplasmic side. To elucidate this mechanism, we have used a combination of MD simulations and both quantum mechanics (QM) and molecular mechanics (MM) free energy calculations. These complementary approaches yield the characteristics of the dynamics and the energetics of the binding of each ligand.

Substrate discrimination could occur at different levels: (i) during migration of the substrate from the periplasm to its binding site and/or (ii) upon binding to the substrate pocket and/or (iii) resulting from the higher affinity of each ligand towards either the aqueous phase or the protein binding pocket (Fig. 1C). In order to investigate these different possible selectivity mechanisms, we first simulated the migration of the three potential substrates (Arg^+^, Arg^2+^, and Agm^2+^) towards the binding site, along the funnel of the transporter in its OF state. Using targeted MD (tMD) simulations, we identified interacting residues that might potentially be essential to guiding the substrate during the course of its translocation. Secondly, we examined the stability of each potential ligand in the binding pocket, using MD simulations. Finally, the relative free energy of binding of each ligand in the binding site pocket was estimated on the basis of both QM and MM free energy calculations, so as to distinguish good from poor binders.

We show that AdiC binds Arg^+^ preferentially to the divalent Arg^2+^ and Agm^2+^ mainly as a result of its lower global charge, although local interactions involving the middle gate aromatic residue (Trp293) and the distal gate residue in its charged form (Glu208^−^) occur more persistently with monovalent Arg^+^. We also find that the protonation state of Glu208 has a strong influence on the binding of the different ligands.

## Results

### Protonation states in the AdiC OF open substrate-free and substrate-bound states

**Figure 2:**
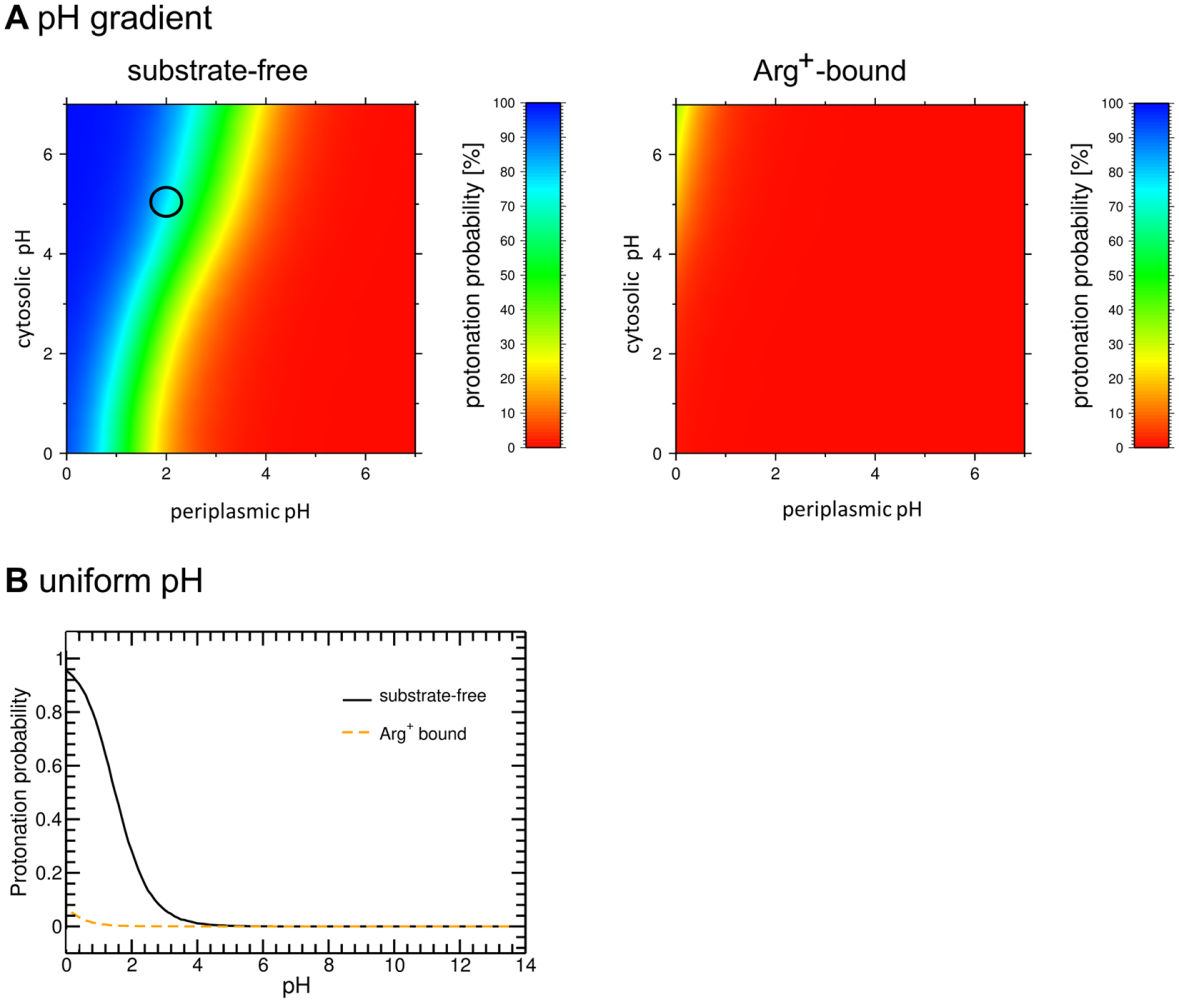
Protonation probability of Glu208. (**A**) Titration curve of Glu208 computed with a pH gradient (two-compartment model) ranging from 0 to 7 on both sides of the membrane in the substrate-free (left) and Arg^+^-bound state (right). The protonation probability is color-coded according to the scale shown on the right. An open back circle indicates the location of pH 2:5 (periplasm:cytoplasm). (**B**) Titration curve of Glu208 computed with a uniform pH for the substrate-free (black solid line) and Arg^+^-bound (orange dashed line) protein in the OF open state (for more details see Material and Methods).

In pH-dependent systems, titratable residues are often pointed out as potential molecular switches involved in protein binding and transport^24,25^. We investigated the influence of a transmembrane pH gradient on the titration behavior of AdiC (see Material and Methods). For the sake of comparison, a similar calculation was performed under uniform pH conditions across the membrane. At a homogeneous pH of 6, at which AdiC still shows respectable transport activity^26^, all ionizable groups including Glu208 are predicted to be in their standard protonation state in both the substrate-free and Arg^+^-bound states. When a pH2:5 (periplasm:cytoplasm) gradient is applied, representative of conditions reigning in the stomach, several periplasmic acidic residues are protonated (Table S1). The protonation probability of Glu208 is fairly high (around 70%) in the substrate-free OF open state, though it is sensitive to slight pH variations (Fig. 2A). In contrast, in the Arg^+^-bound state, Glu208 is very likely to be in its deprotonated form (Fig. 2A), which could suggest its importance in the transport activity AdiC. This dramatic change in Glu208 protonation probability between substrate-free and Arg^+^-bound AdiC is also observed in the Arg^2+^- and Agm^2+^-bound proteins (Fig. S1) and when a uniform pH is simulated (Fig. 2B). This pH dependence suggests that Glu208 forms electrostatic interactions with other titratable groups upon substrate binding. The only new interaction established by Glu208 upon substrate binding is a medium-range (~8 Å) ionic interaction between its carboxylate group and the guanidinium (Gdm^+^) group of each ligand (Fig. S2). This additional interaction supports the deprotonated state of Glu208 predicted by the titration calculations.

### Substrate migration to the binding site in the AdiC OF open state

In order to establish whether selective filtering in favor of Arg^+^ takes place during the migration of the ligand from the periplasm to the binding site along the transporter funnel, nine different tMD simulations of an AdiC dimer were performed (see Material and Methods). All ionizable residues of the membrane-embedded transporter were set to their predicted protonation state for the substrate-free OF open state under a transmembrane pH gradient (pH 2:5). For the sake of comparison, tMD simulations were also carried out with the protonation states predicted for a uniform pH 6 across the membrane. Under these conditions, AdiC is still functional but less effective^26^.

At pH 2:5, the Arg^+^ carboxylate group forms an ionic interaction with Arg256 in the upper part of the funnel that does not occur with Agm^2+^ or Arg^2+^ (Fig. S3). Above the binding pocket, the longer-lasting interactions of all ligands are similar and include cation-π interactions formed by their Gdm^+^ group with the side chains of Trp202 and Phe350. Likewise, the more persistent interactions are similar in the binding pocket for all ligand simulations. They notably include the cation-π interaction formed by the Gdm^+^ with Trp293 located at the bottom of the binding site and H bonds with Ala96 and, to a lesser extent, other polar residues of the pocket. The Arg^+^ carboxylate group and the Arg^2+^ carboxyl group also form several H bonds, in particular with Ser26 and Gly27. These bonds are less persistent for Arg^2+^.

**Figure 3:**
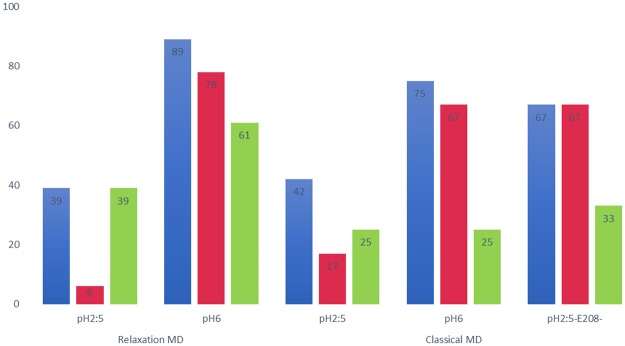
Percentages of ligand occupation in the binding site at the end of the simulations. Percentages of trajectories featuring Arg^+^ (blue), Arg^2+^ (red) or Agm^2+^ (green) in the binding site at the end of the relaxation (left two clusters) and of the conventional MD simulations (right three clusters).

The tMD simulations performed at pH 6 feature some notable differences as compared to the simulations at pH 2:5, mainly due to deprotonation of some of the acidic residues of AdiC (Fig. S4 versus Fig. 3). In the upper part of the funnel, the Gdm^+^ group of each ligand forms ionic interactions with the negatively charged residues Glu349 and less persistently Glu409, in line with the findings of a previous MD study^23^. The amino groups of Agm^2+^ and to a lesser extent of Arg^+^ also form extensive ionic interactions with these two residues. In the binding site, all three ligands form similar interactions to those observed at pH 2:5.

In summary, the interactions formed during migration to the binding site at pH 2:5 are similar for all three ligands, with the exception of a small number of interactions formed by the carboxylate group of Arg^+^ that could contribute to better guiding of this residue toward the binding site.

### Substrate binding to the AdiC OF open state

After simulating their migration along the OF open funnel, we explored the stability of each ligand in the binding site, using relaxation MD trajectories starting from the end of the tMD simulations and conventional MD trajectories (see Material and Methods).

At the end of the pH 2:5 gradient MD simulations, ligand occupancy of the binding pocket is much less frequent for Arg^2+^ than for Agm^2+^, and less frequent for Agm^2+^ than for Arg^+^. At pH 6, the order is Agm^2+^ < Arg^2+^ < Arg^+^, i.e. the rankings of Arg^2+^ and Agm^2+^ are switched (Fig. 3). Overall, Arg^+^ remains bound in the binding pocket at the end of more simulations than Arg^2+^ or Agm^2+^, regardless of the simulation type and pH conditions. Furthermore, the occupancy of the binding site markedly increases with the pH of the simulations (Fig. 3).

**Figure 4:**
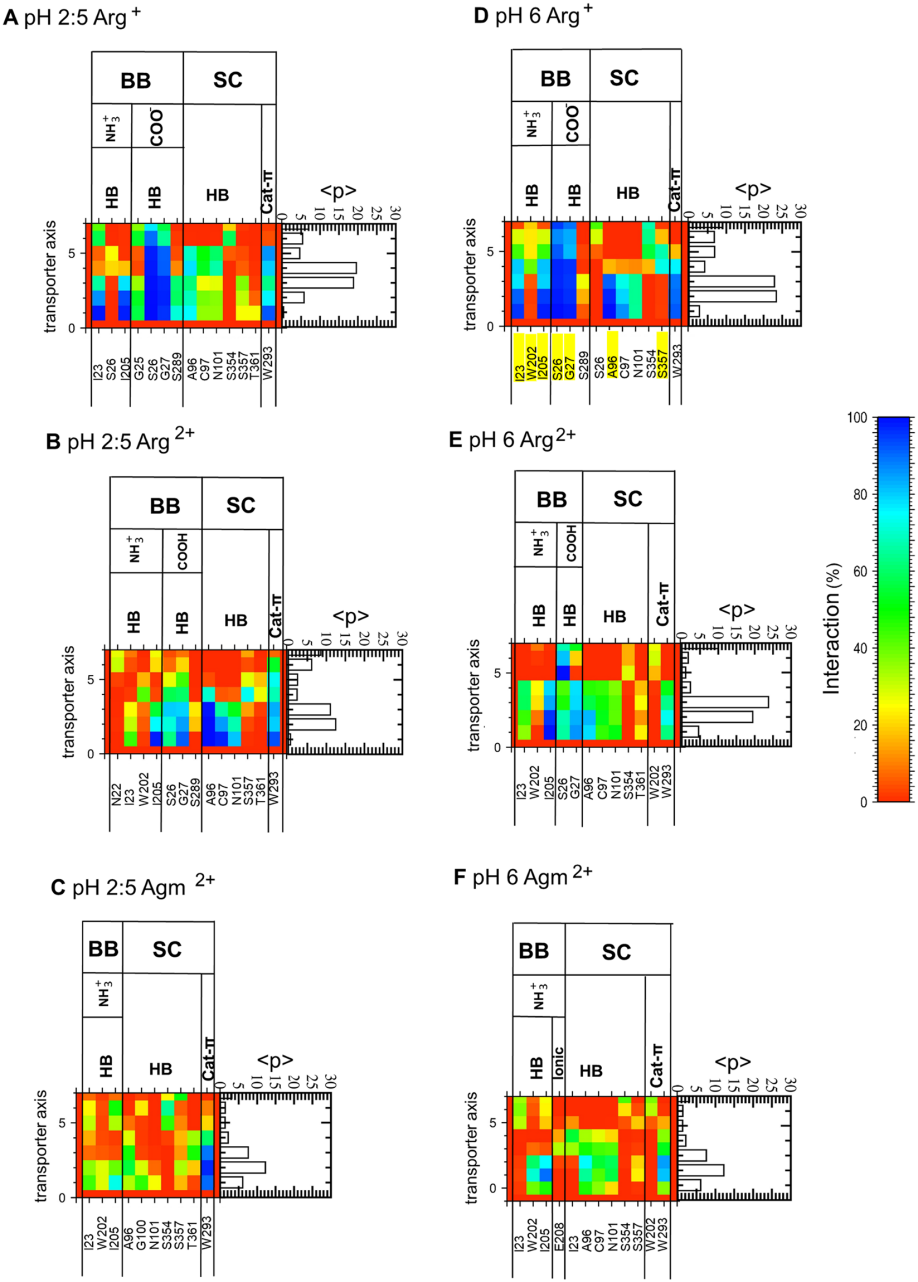
Interactions formed by the ligands in the binding site. H bonds, ionic and cation-π interactions formed at pH 2:5 (**A**–**C**) and pH 6 (**D**–**F**) between the Arg^+^ (**A**,**D**), Arg^2+^ (**B**,**E**), or Agm^2+^ (**C**,**F**) backbone (BB) or sidechain (SC) and protein residues (both BB and SC) in the classical MD simulations. A bar graph of the percentage of the simulations during which the ligands are observed at each transporter axis value is also shown on the right side of the interaction charts. Results are shown for the 12 monomers of the 6 classical MD trajectories. Only interactions occurring in more than 20% of the conformations in at least one bin are shown. Observed interactions between the Arg ligand and the protein residues in the OF open crystal structure are highlighted in yellow in figure (**D**) (PDB ID: 3OB6, Monomer A; Table S2).

In order to explore the possibility of a local ligand discrimination mechanism, we identified the interactions established by the ligands with protein residues in the binding site during the classical MD simulations. Under pH 2:5 gradient conditions (Fig. 4), Arg^+^, Arg^2+^, and Agm^2+^ form almost identical interactions in the binding pocket region (defined as ~0 < z < 6 Å), albeit with varying occupancy. The only exceptions are the interactions of the carboxylate/carboxyl group, which occur only for Arg^+^/Arg^2+^. The carboxylate group of Arg^+^ forms longer-lasting H bond interactions than the carboxyl group of Arg^2+^ with the Ser26 sidechain and with Gly27 (Fig. 4). The Gdm^+^ groups of all three ligands also establish a persistent cation-π interaction with Trp293 that shapes the floor of the binding site (Table 1). Under a pH 6 uniform condition, all three ligands form similar interactions to those observed at pH 2:5 in the binding pocket region, but in contrast to the latter simulations, the cation-π interaction with Trp293 is more persistent with Arg^+^ than with either divalent compound (Table 1). Most of the long-lasting interactions established between the bound arginine and the binding site in trajectories (Fig. 4) are also observed in the OF open crystal structure (Table S2).

**Table 1:** Percentages of specific interactions formed by the Gdm^+^ group of the considered ligand and by Glu208 in the different classical trajectories under varying pH conditions.

Interaction type	Interacting residue	pH2:5			pH 2:5-E208^−^			pH6		
		Arg^+^	Arg^2+^	Agm^2+^	Arg^+^	Arg^2+^	Agm^2+^	Arg^+^	Arg^2+^	Agm^2+^
**Ligand Gdm** ^**+**^										
Cation-π	Trp293	74/−	80/−	87/−	80/−	51/−	58/−	80/−	65/−	64/−
**Glu208 side chain**										
Anion-π	Tyr293	−/−	−/−	−/−	34/5	48/2	48/2	37/6	56/2	39/18
	Trp93	−/−	−/−	−/−	23/96	35/96	35/96	22/95	28/97	25/67

Each pair of numbers (whenever it appears) corresponds to a calculation performed on two portions of the MD trajectories: the first number includes the conformations featuring the ligand in the binding site and the second, those where the ligand is absent from the binding site. This distinction is based on the location of the center of mass of the ligand along the main axis of the transporter (between 0 and 6 Å or beyond 6 Å).

Given the important role of water molecules in mediating protein-ligand interactions, we also examined the hydration degree of each ligand in the binding site. At pH 2:5, Arg^+^ is less hydrated than the divalent ligands (Table S3). The deprotonation of Glu208 reduces the hydration degree of all three ligands.

The fact that ligand unbinding is pH-dependent (Fig. 3) points to the distal gate residue Glu208 being an important factor for binding, since its protonation state depends on the pH and on the presence of a ligand in the binding site (Figs 2 and S1). To study the impact of the Glu208 protonation independently, 6 conventional MD simulations (pH2:5-E208^−^) were performed under pH2:5 conditions with deprotonated Glu208, the form it adopts in the uniform pH6 simulations. These simulations show highly similar features to those observed in the pH6 simulations (Fig. 4D–F), namely similar interactions between the ligands and the protein residues (Fig. S5) and the lesser hydration of Arg^+^ compared to the divalent compounds in the binding site (Table S3). More remarkably, Glu208^−^ increases the stability and residence time of all three ligands in the binding site, as is observed at pH 6 (Fig. 3 and Table S4). All these observations point to a strong influence of the protonation state of Glu208 on ligand binding. This can be explained by the observation during the simulations of both a direct interaction of Glu208^−^ with the ligand and a mediated interaction by another binding site residue. Firstly, Glu208^−^ forms in both the pH2:5-E208^−^ and pH6 MD simulations a direct medium-range ionic interaction (average distance: 8 to 9 Å) with the Gdm^+^ of each ligand in both the pH2:5-E208^−^ and pH6 MD simulations (Table S5). This interaction is also observed in the crystal structures (Fig. 1). The interaction plots (Figs 4, S3, S4 and S5) do not include this ionic interaction since a shorter distance cut-off (6 Å), indicative of short-range ionic interactions, was applied. Secondly, Trp293 plays a mediating role again in both the pH6 and pH2:5-E208^−^ simulations, forming on the one hand an anion-π interaction with Glu208^−^ (with an average distance of about 6.5 Å) and on the other hand a cation-π interaction with the Gdm^+^ group of each ligand (with distances ranging from 3.8 to 4.8 Å) (Table 1). The anion-π interaction occurs mostly in ligand-bound conformations, suggesting possible cooperativity with formation of the cation-π interaction. Interestingly, this cation-π interaction is less persistent in the simulations performed with the divalent ligands and Glu208^−^. In ligand-free conformations, the loss of this anion-π interaction with Trp293 correlates with the formation of a different anion-π interaction formed with Tyr93 (Table 1).

In summary, Arg^+^ resides for a markedly longer time in the binding site than Arg^2+^ or Agm^2+^ in the pH 2:5 simulations (Table S4). This residence time increases upon deprotonation of Glu208. Moreover, the impact of Glu208 on the bound ligand arises from a combination of interactions established when the residue is deprotonated: a direct, medium-range ionic interaction and interactions mediated by the aromatic side chain of Trp293.

### Energetics of substrate binding to the OF open state of AdiC

The selectivity of AdiC towards Arg^+^ over Arg^2+^ or Agm^2+^ could be due to Arg^+^ having a higher affinity for the AdiC binding site than the periplasmic medium. We thus used two different approaches to evaluate the free energy of binding of the three potential ligands based either on QM or MM calculations both combined with an implicit treatment of the solvent.

MM-PBSA is an attractive approach in which a conformational sampling is performed. This method has been used successfully to reproduce experimental data^27^. However, because most molecular-mechanics force fields have a limited accuracy, we also applied a QM-cluster approach in which a small model of the most important residues is cut from the binding site and is studied in a continuum solvent, here for two conformations extracted from each monomer^28^. In this QM procedure the free energy of binding (ΔG_binding,solv_) was calculated for each ligand from the gas-phase interaction energy ΔE_int_ and from the solvation free energies of the ligand (ΔG_solv,ligand_), protein (ΔG_solv,p_) and complex (ΔG_solv,complex_) (see Materials and Methods for detail and equations).

The computed gas-phase ΔE_int_ values in the binding pocket indicate that in a vacuum, Arg^+^ binds AdiC less favorably than Agm^2+^ or Arg^2+^, regardless of the AdiC monomer or the protonation state of Glu208. The two divalent ligands show similar interaction energies (Table 2). Protonation of Glu208 reduces ΔE_int_, with a stronger effect on the divalent ligands (about 60 to 70 kcal/mol) than on monovalent arginine (about 35 to 40 kcal/mol) (Table 2). A stronger reduction in ΔE_int_ is also found for monomer B than for monomer A, probably because the Gdm^+^ group of the ligand points away from the binding pocket towards the solvent in monomer B (Table 2).

**Table 2:** Contributions to the free energies of binding calculated using a QM procedure.

Structure	Glu208 charge	Arg^+^				Agm^2+^				Arg^2+^			
		ΔE_int_	ΔG_solv,ligand_	ΔG_solv,p_ polar	ΔG_solv,complex_ polar	ΔE_int_	ΔG_solv,ligand_	ΔG_solv,p_ polar	ΔG_solv,complex_ polar	ΔE_int_	ΔG_solv,ligand_	ΔG_solv,p_ polar	ΔG_solv,complex_ polar
A	−1	−137.8	−113.5	−106.8	−103.7	−191.0	−190.8	−106.8	−114.8	−196.8	−197.7	−106.8	−113.9
B	−1	−80.6		−105.1	−105.2	−131.0		−105.1	−122.9	−139.2		−105.1	−123.0
A	0	−95.9		−86.6	−108.7	−122.4		−86.6	−143.2	−130.4		−86.6	−141.2
B	0	−47.9		−85.3	−110.8	−67.6		−85.3	−148.6	−75.8		−85.3	−149.0

Gas-phase interaction energies (ΔE_int_) and polar contributions to the solvation free energies (ΔG_solv,complex_, ΔG_solv,p_, ΔG_solv,ligand_) for Arg^+^, Arg^2+^ and Agm^2+^.

In contrast, the solvation free energy component ΔG_solv,ligand_ opposes ligand binding (Eqs 1 and 2). As expected, this term is significantly smaller for monovalent Arg^+^ than for divalent Agm^2+^ or Arg^2+^ (by about 80 kcal/mol). The solvation free energies of monoatomic multivalent ions are known to be subject to higher errors than those of monovalent ions, though in the divalent ligands considered here, the total +2 charge is not locally concentrated as it is in monoatomic multivalent ions but is distributed between different locations on the molecules^29^. The solvation free energy components of the complex (ΔG_solv,complex_) and of the protein (ΔG_solv,p_) are fairly high and positive. This clearly arises from the apolar cavitation term (Table S6). This term is similar for all ligand-bound complexes and will therefore cancel out when the ligands are compared. The polar contributions to ΔG_solv,complex_ and ΔG_solv,p_, both of which are negative, respectively favor or oppose ligand binding (Eqs 1 and 2). These components are sensitive to the total charge of the system, to which the charge of Glu208 and that of the ligand contribute (Table 2).

Thus, the difference in solvation of the free ligand favors Arg^+^ binding to AdiC over Agm^2+^ or Arg^2+^ by about 70 to 90 kcal/mol, whereas the difference in solvation of the complex favors binding of the divalent ligands by about 10 to 30 kcal/mol, depending on the protonation state of Glu208 (Table 2). Since the solvation free energy of the unbound protein, ΔG_solv,p_, cancels out between different ligands, the total ΔΔG of solvation (Eq. 2) favors Arg^+^ binding by about 40 to 60 kcal/mol (Table 3).

**Table 3:** Free energies of binding calculated using a QM procedure.

Structure	Glu208 charge	Agm^2+^			Arg^2+^		
		ΔΔE_int_	ΔΔG_solv_	ΔΔG^*^_binding_	ΔΔE_int_	ΔΔG_solv_	ΔΔG^*^_binding_
A	−1	−53.2	66.2	13.0	−59.0	73.9	1.9
B	−1	−50.4	59.6	9.2	−58.6	66.5	7.9
A	0	−26.5	42.8	16.3	−34.5	51.7	17.2
B	0	−19.7	39.5	19.8	−27.9	46.0	18.1

Difference in binding free energy without configurational entropy (ΔΔG^*^_binding_), difference in total solvation energy (ΔΔG_solv_), and difference in gas-phase interaction energy (ΔΔE_int_) (Eq. 3) for Arg^2+^ and Agm^2+^ with respect to Arg^+^ used as a reference. All energies were computed for both monomers (A and B) and both Glu208 protonation states in 3OB6. All energy values are given in kcal/mol.

The entropy changes upon ligand binding (−TΔS) are notoriously difficult to estimate. However it has been shown in some cases that neglecting the entropy contributions yields accurate enough predictions when comparing the binding of different ligands^27^. The difference in binding free energy, ΔΔG^*^_binding_, which lacks configurational entropy contributions, indicates that Arg^+^ binds AdiC more strongly than Arg^2+^ or Agm^2+^ regardless of the monomer and that this difference increases with Glu208 protonation. This preferential driving of Arg^+^ into the AdiC binding pocket arises mainly from the energetically unfavorable breaking of the water-ligand interactions, which are stronger with the divalent ligands. This preferential driving of Arg^+^ into the AdiC binding pocket arises mainly from the strong energy penalty for stripping water molecules from Agm^2+^ or Arg^2+^ as compared to Arg^+^.

To nevertheless assess the impact of the neglected entropy contributions, translational, rotational and vibrational entropies were evaluated upon ligand binding using the standard rigid rotor/harmonic oscillator approximation (Table S7A). We also estimated the conformational entropy contribution from the number of rotatable bonds hindered upon complex formation by applying a penalty of 1 kcal/mol per bond (Table S7B). The largest entropy difference arises from the conformational entropy which is about +/−1 kcal/mol between the divalent and monovalent ligand. This being said, the inclusion of the different entropy terms does however not change the ranking in binding free energy of the monovalent and divalent compounds (Table S7C).

The binding free energies for Arg^+^, Arg^2+^, and Agm^2+^ were also computed with a hybrid MM and continuum solvent method, MM-PBSA (see Materials and Methods).

Similarly to what was found in the QM calculations, the MM-PBSA gas phase interaction energy ΔE_int_ (Eq. 1) favors binding of the three ligands regardless of the pH. ΔE_int_ decreases with Glu208^0^, and the reduction is greater for the divalent ligands (Table 4). In contrast to the QM calculations, the MM-PBSA calculations do not predict that the two divalent compounds, in particular Agm^2+^, will be the strongest binders in a vacuum. This is likely due to the use of different trajectories for each ligand, whereas the QM calculations were carried out on a single conformation, optimized for each ligand, meaning the binding site geometries are almost identical as for the protein part (Material and Methods). The QM ΔE_int_ variations should therefore arise mostly from the different electronic distributions on the different ligands. This explains both the significantly more favorable ΔE_int_ values for the divalent ligands and the reduction of ΔE_int_ upon protonation of Glu208. Also in contrast to the QM calculations, the MM-PBSA ΔE_int_ values include, in contrast to the QM values, the dynamics of both the transporter and the ligand, which appears to significantly influence binding in the protonated Glu208 simulations (Table 4).

**Table 4:** Contributions to the free energies of binding calculated using MM-PBSA.

Structure	Glu208 charge	Arg^+^				Agm^2+^				Arg^2+^			
		ΔE_int_	ΔG_solv,ligand_	ΔG_solv,p_ Polar	ΔG_solv,complex_ polar	ΔE_int_	ΔG_solv,ligand_	ΔG_solv,p_ Polar	ΔG_solv,complex_ polar	ΔE_int_	ΔG_solv,ligand_	ΔG_solv,p_ polar	ΔG_solv,complex_ polar
A + B	−1	−134.2	−102.6	−82.9	−79.0	−134.6	−187.4	−80.9	−96.6	−162.41	−186.4	−21.7	−94.0
A + B	0	−85.6	−97.2	−55.6	−80.1	−47.0	−186.6	−55.4	−126.7	−75.0	−185.7	−54.7	−119.9

ΔE_int_, ΔG_solv,complex_, ΔG_solv,p_, and ΔG_solv,ligand_ for Arg^+^, Arg^2+^, and Agm^2+^.

In keeping with the QM data, the free energy of solvation of the ligands (Eqs 1 and 2) calculated with MM-PBSA opposes binding to AdiC, with a much higher penalty for the divalent ligands. Likewise, the apolar solvation contributions to ΔG_solv,complex_ and ΔG_solv,p_ are almost identical for all three ligands and should not be discriminating contributions (Table S6). Their polar contributions are negative and should thus either favor (in the complex) or oppose (in the protein alone) the binding of all three ligands (Eqs 1 and 2).

The MM-PBSA free energy of binding in solution, ΔΔG^*^_binding_, assuming the entropy terms to be similar and to cancel out when comparing the potential ligands, indicates that Arg^+^ binds to AdiC preferentially compared to Arg^2+^ or Agm^2+^ and that Glu208^0^ increases this preference (Table 5), in agreement with the QM results. This arises from the difference in ligand hydration free energy between monovalent Arg^+^ and the divalent ligands and from the difference in gas phase interaction energy ΔE_int_, which does not favor the divalent compounds (in contrast to what the QM data indicates), particularly with the neutral form of Glu208.

**Table 5:** Free energies of binding calculated using MM-PBSA.

Structure	Glu208 charge	Agm^2+^			Arg^2+^		
		ΔΔE_int_	ΔΔG_solv_	ΔΔG^*^_binding_	ΔΔE_int_	ΔΔG_solv_	ΔΔG^*^_binding_
A + B	−1	−0.4	65.2	61.8	−28.2	67.7	39.4
A + B	0	38.6	42.6	81.2	10.6	47.8	58.4

Difference in binding free energy not including the configurational entropy contribution (ΔΔG^*^_binding_), total solvation energy (ΔΔG_solv_), and gas-phase interaction energy (ΔΔE_int_) for Arg^2+^ and Agm^2+^, with respect to Arg^+^ used as a reference. All energies were averaged for the two monomers (A and B) and computed for both Glu208 protonation states. All energy values are given in kcal/mol. Standard errors are given in Table S8.

The vibrational entropy term to the MM-PBSA ΔΔG^*^_binding_ energy was estimated using a quasi-harmonic analysis (Table S7D). Accounting for this contribution does not reverse the hierarchy of the monovalent versus divalent ligand affinity (Table S7E).

## Discussion

The amino acid antiporter AdiC plays a key role in an acid resistance mechanism of *E*. *coli*, by linking the proton-consuming decarboxylation of arginine to agmatine to the exchange of intracellular agmatine for extracellular arginine. To promote proton extrusion exclusively, AdiC should transport only one of the two major protonated forms of arginine present under the acidic conditions prevailing in the stomach: the singly protonated Arg^+^^11^. We have used MD simulations and both MM and QM calculations to infer a potential microscopic-level discrimination mechanism explaining the preferential uptake of Arg^+^ compared to Arg^2+^ as well as Agm^2+^. This mechanism is mainly based on the total charge carried by each ligand, as was previously proposed^11^, but it also relies on specific local interactions modulated by an acidic residue lining the bottom of the binding site. Furthermore, the protonation state of this residue plays a crucial role in the selectivity mechanism.

### Insights into how AdiC might distinguish Arg^+^ from Arg^2+^ along the funnel and in the binding site

The major differences in local interactions between Arg^+^ and Arg^2+^ occur mainly in the binding pocket. Through its carboxylate group, Arg^+^ forms more persistent H bonds than Arg^2+^ with Ser26 and Gly27, regardless of the pH conditions tested (Fig. 4). It has been proposed that Ser26 does not govern ligand selectivity through sidechain H bond interactions, since a fully transport-active AdiC S26A mutant shows a normal capacity to distinguish Arg^+^ from Arg^+2^ ^11,30^. However experimental and theoretical studies, however, have shown that water molecules can, in some cases, take over the role of the mutated residue within the binding pocket^31,32^. One can therefore not totally rule out the possibility that the Ser26 sidechain or a replacing bound water molecule might act in a local mechanism. As for Gly27, its potential importance is supported by comparison with the monoamine transporters adopting the same fold as AdiC. In these transporters, the side chain carboxylate of an aspartate occupying the same backbone position in the binding site as Gly27 in AdiC occupies an equivalent position to that of the Arg^+^ carboxylate^33^.

In simulations with protonated Glu208^0^, likely to be the prevalent form under acidic conditions, Arg^+^ differs from both Arg^2+^ and Agm^2+^ by a more stable position and a longer residence time in the binding site region as well as less ligand hydration (Tables 1 and S4). This suggests that the discrimination among the ligands could be driven first by the Glu208^0^. The observed larger hydration degree is in line with a recent study proposing hydration of Agm^2+^ as a factor favoring its disengagement from the binding site^22^. The Glu208^**−**^ form increases the stability and residence time of all three ligands in the binding site (Tables 1 and S4) and reduces their hydration (Table S3). This highlights the impact of the medium-range attractive electrostatic interaction established between Glu208^**−**^ (whose protonation state changes upon ligand binding, see Fig. 1) and the positive charge of the ligand on its stabilization in the binding site.

Another effect of Glu208^**−**^ is that it diminishes the persistence of the cation-π interaction between the Gdm^+^ group of the divalent ligands and the middle gate residue Trp293 whereas it increases this interaction persistence for monovalent Arg^+^ (Table 1). This gating residue is known to be crucial for AdiC activity^19,20,34^ (Table 1). The decreased persistence of this interaction in the case of Arg^2+^ and Agm^2+^ correlates with their shorter residence time. Trp293 is also involved in an in-plane anion-π interaction with Glu208^**−**^, observed primarily in ligand-bound conformations and thus concomitantly with the occurrence of its cation-π interaction with the ligand. In contrast, ligand binding induces the loss of another anion-π interaction formed by Glu208^**−**^ and Tyr93 (Table 1). Contacts between an anion and aromatic residues have recently been recognized as an important type of noncovalent bonding interaction in biological processes^35,36^. They have been detected in protein structures^35^ with a preferred close-to-parallel orientation, which is the arrangement found in our study. Furthermore, a large number of anion–π contacts have been found to be engaged in triads with a cation forming an interaction with the aromatic group^35^. This again is observed in our simulations of the substrate-bound state. Thus, Glu208^**−**^ together with Trp293 are involved in intertwined interactions established upon ligand binding, preferentially with monovalent Arg^+^ (Fig. S2). Those two residues have been reported to be crucial for substrate transport as substitutions into Ala and Asp for Glu208 and into Leu for Trp293 led to severely compromised transport^12,26^. Trp293 could play a pivotal role by forming an interaction with both the guanidinium of Arg^+^ and the carboxylate anion of Glu208 with its aromatic side chain.

### Charge-based discrimination of Arg^+^ versus Arg^2+^ and Agm^2+^

On the basis of the contributions to the free energy of binding estimated here by means of MM-PBSA and QM calculations, it seems that the ability of AdiC to discriminate against either Arg^2+^ or Agm^2+^ in favor of Arg^+^ is driven mainly by the greater degree of hydration of divalent ligands resulting from their total charge, as suggested by Tsai and Miller^11^. Our MM-PBSA results (obtained from a set of conformations differing for each ligand instead of a single protein-ligand conformation as in the QM calculations) additionally suggest that the interaction energy ΔE_int_ could favor binding of the monovalent Arg^+^ in simulations including Glu208^0^.

Most remarkably, our MM-PBSA and QM estimates concur to predict that Glu208^0^, probably the most abundant form of the residue given the acidity of the extracellular medium, further destabilizes the two divalent ligands relative to Arg^+^. This again hints at a key role played by Glu208 in ligand stability in the binding pocket as well as in ligand discrimination. Moreover, monovalent arginine, which has the highest affinity, shows the lowest number of unbinding events in contrast to the divalent ligands suggesting that the ligand specificity might be related to the dissociation constant^37^.

MM-PBSA and QM-PCM or QM-SMD methods have their own limitations as with any method. Inaccuracies inherent in MM-PBSA may arise from the use of implicit solvent and the choice of the dielectric constant, from uncertainties in the calculations of entropic terms and inaccuracies in the force field^27^. For example, a study has pointed to the inability of the Charmm36 force field to accurately reproduce interactions with Ca^2+^ although it reasonably describes the interactions between monovalent ions and several metalloproteins^38^. Even though two out of our three ligands are divalent cations their total charge is not, in contrast to Ca^2+^, carried by a single atom but distributed in the form of two single charges themselves distributed as partial charges over several atoms. However, because of these potential weaknesses of classical force fields we also used a QM method to estimate the free energy of binding.

Regarding the QM calculations, uncertainties depend on the assumptions of the method used, HF and/or DFT. The use of a single conformation for the ligand and two for the complex and the empty protein is also a source of possible errors. These compromises had to be made because of the high computing time cost. As in MM-PBSA, the QM continuum solvation models are also error-prone^39^.

Argininamide, an analogue of Arg^2+^, was shown, in binding experiments monitoring the exchange in liposomes of unlabeled argininamide with radiolabeled Arg, to be a poor transport substrate^34,40^ with a 30-fold higher concentration required to mimic competition by Arg^34^. This difference in the inhibition constant (corresponding to a free energy difference of about 2 kcal/mol) can be compared to the difference in free energy of binding between divalent and monovalent arginine computed from the QM (Table S7C) and the MM-PBSA calculations (Table 7E) of 3 and 52 kcal/mol respectively, for the unprotonated Glu208 consistent with the pH value of about 6 of the binding assays. The inaccuracies inherent to the two methods, mentioned above, may explain the discrepancies between the experimental and theoretical values though the QM calculations provide, in this case, a much better estimation. We however think that the QM and MM results which feature a similar trend buttress our proposed mechanism of AdiC selectivity.

### Chemical features involved in the discrimination of Arg^+^ versus Arg^2+^ and Agm^2+^

**Figure 5:**
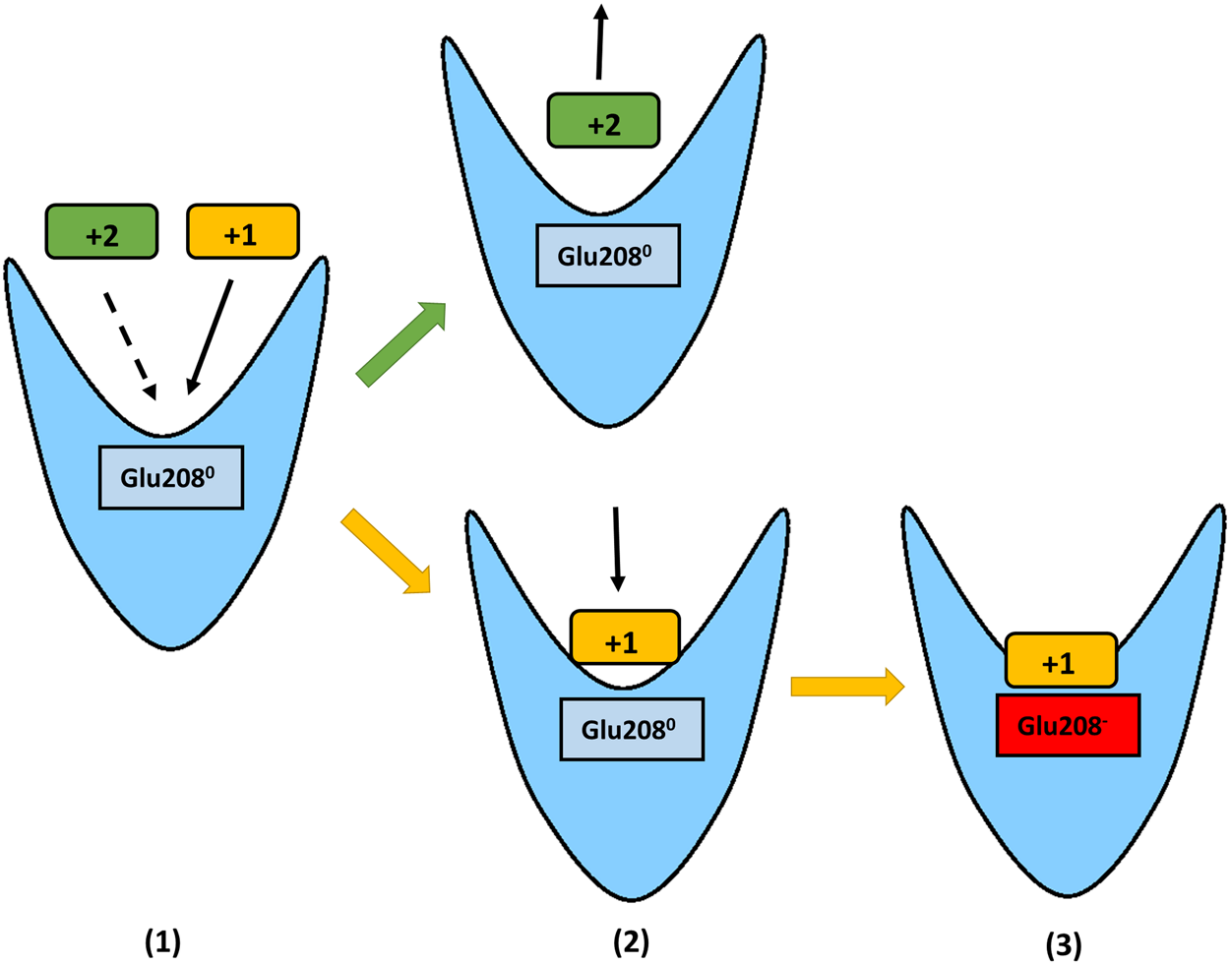
Schematic overview of ligand selection by the AdiC binding site. (**1**) Under acidic conditions, during migration of the divalent (green box) or monovalent (orange box) ligand to the binding site, Glu208^0^ (light blue rectangle) remains protonated. Note here that migration should be less likely for the divalent compounds since their affinity for the aqueous periplasm is higher than that of Arg^+^ as suggested by the dashed arrow. (**2**) Glu208^0^ favors tight binding of monovalent Arg^+^ and drives away divalent Arg^2+^ and Agm^2+^, which remain more hydrated and are released on the outside. (**3**) Arg^+^ enters more deeply into the binding site and shields Glu208 from the acidic medium. This promotes the deprotonation of Glu208, followed by tighter binding of Arg^+^, which then forms specific direct and mediated interactions.

Several lines of evidence point to the key role of Glu208 in ligand binding and discrimination. Remarkably, Glu208 protonation influences both the free energies of binding calculated for the singly and doubly charged ligands (Tables 3 and 4) and the persistence of local interactions at the level of the binding site (Fig. 4 and Table 1). From our simulations and calculations, we infer a series of events which rationalizes the discrimination of monovalent Arg^+^ from divalent Arg^2+^ or Agm^2+^ and is depicted in Fig. 5. During the migration of a ligand to the binding site, Glu208 is likely to be in its neutral form because of its accessibility to the high H^+^ concentration of the outside medium. The presence of Glu208^0^ significantly reduces the residence time of Arg^2+^ and Agm^2+^ in the binding site compared to Arg^+^ since the divalent ligands have a stronger affinity for the aqueous medium and possibly a lower interaction energy with Glu208^0^. The presence of Glu208^0^ therefore favors unbinding of Arg^2+^ and Agm^2+^ but not of monovalent Arg^+^. The bound Arg^+^ in turn protects Glu208^0^ from the acidic medium and induces the loss of its carboxyl proton. The resulting Glu208^**−**^ further promotes tighter binding of Arg^+^, which is stabilized both by direct ionic and cation-π interactions and by mediated anion-π interactions (Table 1).

In conclusion, our data indicates that the binding site of AdiC discriminates between ligands mainly on the basis of their full charge, in agreement with^11^. Nonetheless, local interactions in which Glu208 plays a pivotal role favor the binding of monovalent Arg^+^. This detailed understanding of the discrimination capacity of AdiC may be important in understanding the selectivity mechanisms of other acid resistance antiporters. It also offers insights into potential strategies for designing new drugs against enteropathogenic bacteria. This is of particular importance, given that food-borne infections caused by bacteria pose a particular risk to human health and have a high cost in terms of medical treatment and lost productivity^41–43^.

Our study opens prospects for understanding the selectivity mechanisms of other transporters of the APC superfamily, particularly eukaryotic APC transporters for which structural determinations have proved very difficult.

## Materials and Methods

### Molecular Dynamics simulation details and protocols

All MD simulations were carried out on dimers of the transporter because of their physiological relevance^26^. We studied isothermal-isobaric ensembles at 300 K with the program NAMD2.9 software^44^ as described previously^23^. The CHARMM27 force field^45^ with CMAP corrections^46^ was used to describe protein, water, and ion atoms. A united atom force field described the lipids^47^.

Binding of Arg^+^, Arg^2+^, and Agm^2+^ to the OF open unbound state was simulated by targeted MD (tMD). tMD simulations were performed because a previous study has shown that conventional simulations failed to produce migration of Arg^+^ to the binding site^23^.

5-ns long tMD simulations during which the arginine substrate was guided to the binding site (using the position of Arg in the OF open arginine-bound structure (PDB ID: 3OB6^19^) as a target) were carried out following a protocol described elsewhere in detail for Arg^+^ binding^23^ under two different pH conditions: a transmembrane pH gradient (pH 2:5) and a uniform pH 6. Three different starting conformations of the protein were extracted from a previous simulation of the OF open unbound state and three initial positions of each substrate randomly positioned at the mouth of the OF open funnel were used. This resulted in a total of 9 binding trajectories for each monomer of the simulated dimers. Each tMD trajectory was followed by a 20-ns conventional simulation to relax the system.

In addition, six 20-ns-long classical MD simulations were performed, starting from the OF open substrate-bound crystal structure^19^ in two pH conditions: a transmembrane pH gradient with either the neutral (pH2:5) or the charged form (pH2:5-E208^−^) of Glu208 and a uniform pH 6. Details of the equilibration protocol with either Arg^+^, Arg^2+^, or Agm^2+^ are given elsewhere^23^. All ionizable groups were set at their protonation states describing two different pH conditions (see below).

### Analysis of the MD simulations

The percentage of trajectories featuring the substrate in the binding site at the end of the MD simulations is given by the number of conformations, at the end of the MD, featuring the center of mass of the ligand in the binding site region (between 0 and 6 Å) divided by the total number of simulations (12 and 18 for the classical MD and tMD simulations, respectively).

The different types of interactions between the ligand and protein residues were monitored in all trajectories and crystal structures using VMD^48^ (for ionic interactions) and EUCB^49^ (for cation-π interactions and direct hydrogen bonds). Interactions are calculated on the basis of criteria published in detail elsewhere^23^. The probability of an interaction being formed by the ligand was estimated along the main axis of the transporter in 1-Å-thick slices. The interaction probability was calculated as the number of snapshots featuring a given interaction and having the center of mass of the ligand heavy atoms located in a given slice divided by the total number of snapshots having the center of mass of the ligand heavy atoms located in the same slice. In the interaction plots the interactions formed with a probability higher than or equal to 20% over all MD trajectories in at least one slice along the main axis are shown.

Anion-π interactions potentially formed by the Glu208 carboxylate group with surrounding residues were computed with an in-house modified version of the program EUCB. A distance smaller than 6 (Tyr, Phe) or 7 Å (Trp) between the centers of mass of the Glu carboxylate sidechain group and of the ring atoms of the aromatic residues in addition to an angle <60° between the plane formed by the Glu carboxylate sidechain group and the ring atoms of the aromatic residues was used. H bonds between Glu208 and other protein residues were identified using the same criteria as for the ligand H bonds.

### Protonation probability calculations

Prior to the protonation probability simulations, the missing loop (residues 345–350) in monomer A of the OF open arginine-bound structure of AdiC^19^ was constructed with MODELLER^50^, using the loop in monomer B as template. A 10-ns MD simulation was then performed on the protein embedded in a lipid environment with an orientation proposed by OPM^51^ and solvated by water and ions with CHARMM GUI^52^ and keeping the protein fixed except for the modeled loop. The protonation probabilities of all titratable groups were determined on the final configuration of this simulation, using the MEAD and GMCT programs^53^. Two different pH conditions were considered. First, a pH gradient across the membrane was mimicked. For this purpose, we used a formalism developed for the calculation of protonation probabilities which takes a pH difference between two reservoirs of protons separated by a membrane into account^53,54^. To simulate a membrane environment, the protein was oriented as proposed by OPM and the membrane hydrophobic core was modeled by a slab ranging from −15 to +15 Å. The headgroup region (5 Å on each side of the membrane) and the membrane hydrophobic core were assigned dielectric constants of 20 and 4, respectively. The hydrophilic cavities and the OF funnel leading from the periplasm to the binding site were filled with water molecules. The location of each titratable residue was ascribed either to the periplasmic or the cytosolic pH region (Table S9). Secondly, a uniform pH was simulated. In both calculations, dielectric constants of 4 and 80 were used for the protein and water, respectively. The temperature was set to 298.5 K and a KCl concentration of 0.1 M was used.

### Binding free energy calculations

#### Quantum calculations

Four different systems were prepared for each ligand according to the following protocol: starting from the OF open crystal structure, residues within a distance of 4.5 Å from the arginine ligand were selected in both monomers A and B. This region contains around 500 atoms and includes Asn22, Ile23, Met24, Gly25, Ser2, Gly27, Ala96, Cys97, Gly100, Ala103, Met104, Trp202, Ser203, Phe204, Ile205, Glu208, Tyr239, Ser289, Trp293. Suitable ACE and/or CT3 patches were applied to terminal residues. This selection also includes Glu208, whose sidechain is considered in two different protonated states. The crystal position of arginine was used as an initial position for Arg^+^, Arg^2+^, and Agm^2+^. Overall, twelve systems were energetically minimized for 100 steps using the CHARMM program^55^ and the CHARMM27 force field.

The free energies of binding of Arg^+^, Arg^2+^, and Agm^2+^ in the AdiC binding site were calculated on the basis of the scoring function developed by Hobza *et al*.^56^.

1 $${\rm{\Delta }}{G}_{binding}={\rm{\Delta }}{E}_{int}+{\rm{\Delta }}{G}_{solv}-T{\rm{\Delta }}S$$

where ΔE_int_ is the interaction energy calculated in a vacuum at the PBE0/cc-pVTZ level, using the BSSE counterpoise methodology to correct for basis set superposition errors. Empirical dispersion corrections were added and calculated with the DFT-D3 program^57^.

ΔG_solv_ is the change in solvation free energy occurring upon complex formation, calculated as follows:

2 $${\rm{\Delta }}{G}_{solv}={G}_{solv,complex}-{G}_{solv,p}-{G}_{solv,ligand}$$

The contributions to the free energies of solvation of the unbound ligands Arg^+^, Arg^2+^ and Agm^2+^ were calculated at the HF/6–31G(d) level using the SMD model^39^ on the PBE0/cc-pVTZ optimized structures. These calculations were performed with Gaussian09 default settings^58^. ΔG_*solv*,*p*_ and ΔG_*solv*,*complex*_ were calculated with the IEF-PCM solvent model at the HF/6–31 + G(d) level with the Gaussian03 default settings and united atom Hartree Fock (uahf) radii^39^. The dielectric constants used to evaluate ΔG_*solv*,*ligand*,_ ΔG_*solv*,*p*_ and ΔG_*solv*,*complex*_ were set to 80, 4 and 4 respectively. The latter two values were chosen because the solvation process in this case consists in transferring the subset of residues lining the binding site with/without the ligand from vacuum to the surrounding rest of the protein and membrane.

We first assumed the entropy terms to be similar and to cancel out when comparing the potential ligands. With this assumption the difference in ΔΔG_binding_ between different ligands thus reduces to:

3 $${{\rm{\Delta }}{\rm{\Delta }}G}_{binding}^{\ast }={{\rm{\Delta }}{\rm{\Delta }}E}_{int}+{\rm{\Delta }}({{\rm{\Delta }}G}_{solv,complex}-{{\rm{\Delta }}G}_{solv,p}-{{\rm{\Delta }}G}_{solv,ligand})$$

Secondly, the change in configurational entropy ΔS_config_ (vibrational, rotational and translational) of the ligands upon binding was estimated in the rigid rotor, harmonic oscillator (RRHO) approximation to the minimized free and bound systems using the MacroModel Embrace module of Schrödinger software with the OPLS-2005 force-field.

#### MM-PBSA calculations

The binding free energies for Arg^+^, Arg^2+^ and Agm^2+^ were computed using a hybrid between MM and a continuum solvent method, MM-PBSA. The relative binding free energy of the complex of AdiC with Arg^+^, Arg^2+^ or Agm^2+^ was computed with Eqs (2) and (3). It was calculated with the g_mmpbsa tool^59^ on the set of residues defined for the QM calculations extracted from portions of the MD trajectories containing conformations featuring the ligand in the binding site under the pH 2–5 conditions with the neutral (pH2:5) and the charged form (pH2:5-E208^−^) of Glu208.

The non-polar solvation contribution to the solvation free energy was evaluated on the basis of the SASA model^27^. The dielectric constants of the corresponding surrounding media were set at the same values as in the QM calculations.

The vacuum potential energy E_int_ here only includes the energy of nonbonded interactions calculated on the basis of the CHARMM27 force field, as the single trajectory approach was used, in which the protein and ligand conformations are identical in the bound and unbound forms.

Like in the QM calculations, the configurational entropy was at first not included in our binding energy calculations ΔΔG^*^_binding_ since it was assumed to be similar for all three ligands and thus not significant in ranking the binding affinities of the substrates^27^. In a second step, configurational entropies were estimated from the variance-covariance matrix calculated with the Gromacs g_covar and g_anaeig commands on MD trajectories. The protein-ligand complex trajectories contained conformations featuring the ligand in the binding site in the pH 2–5 gradient condition with the two protonated forms of Glu208. 40 and 5-ns MD simulations of the protein (with two different protonation states of Glu208) and of each ligand in water were performed, respectively, prior to the entropy calculations.

## Electronic supplementary material

Supplementary Information
