# Supplementary material for: Molecular mechanism of substrate selectivity of the arginine-agmatine Antiporter AdiC
Source: Sci Rep. 2018 Oct 23;8:15607. doi: 10.1038/s41598-018-33963-1 (PMC6199258; doi:10.1038/s41598-018-33963-1)
Supplement: Supplementary file 1 — Supplementary Information [file 41598_2018_33963_MOESM1_ESM.docx]

Title: Molecular mechanism of substrate selectivity of the arginine- agmatine Antiporter AdiC

Short Title: Substrate Selectivity of AdiC

Authors: Eva-Maria Krammer, Andrew Gibbons, Goedele Roos, and Martine Prévost


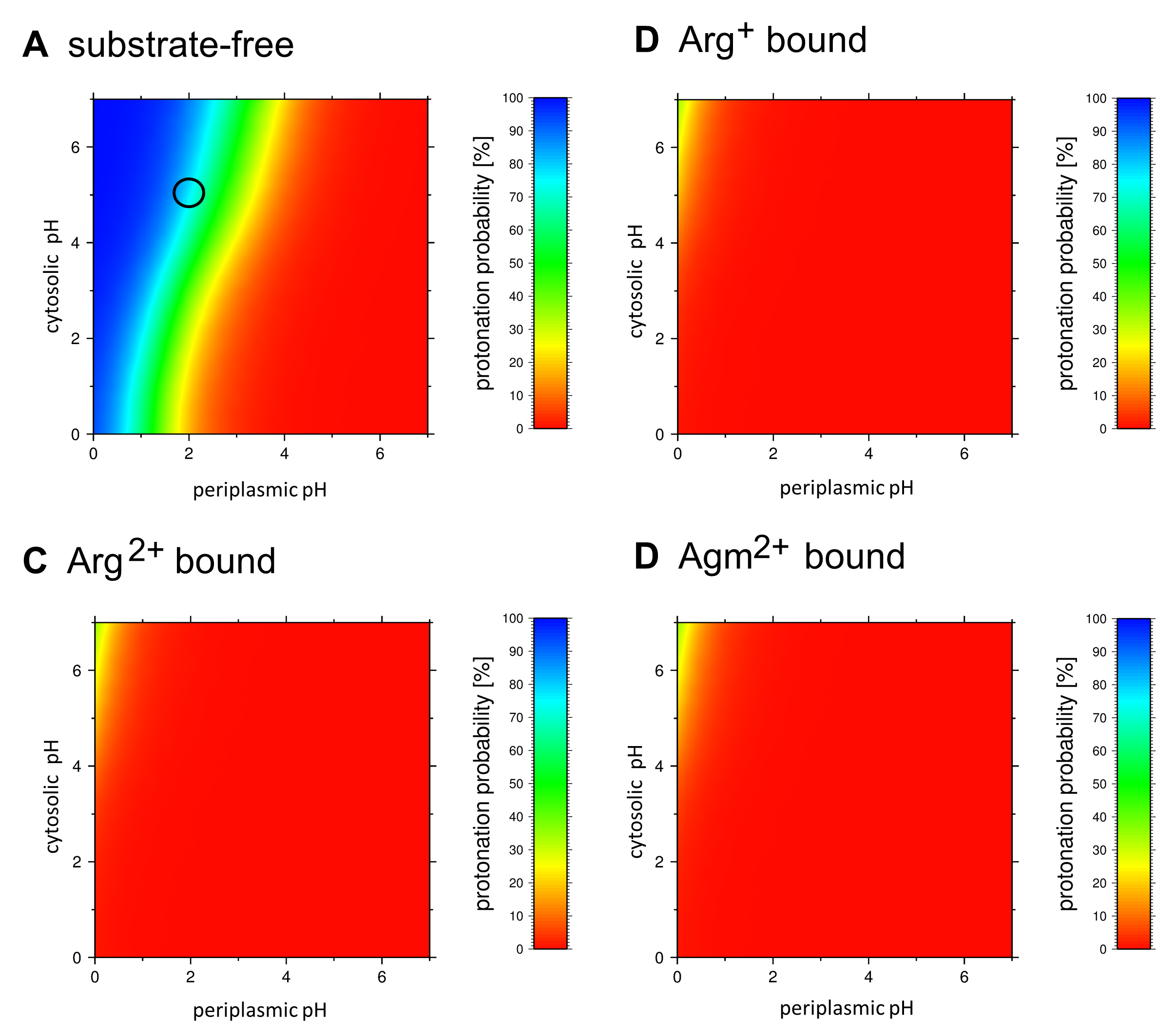


Fig. S1: Protonation probability of Glu208 computed in a pH gradient (two compartment model) ranging from 0 to 7 on both membrane sides in the substrate-free (A) and Arg^+^-bound (B), Agm^2+^-bound (C) and Arg^2+^-bound state (D). The protonation probability is color-coded according to the scale shown on the right. An open back circle indicates the location of pH2:5 in A.


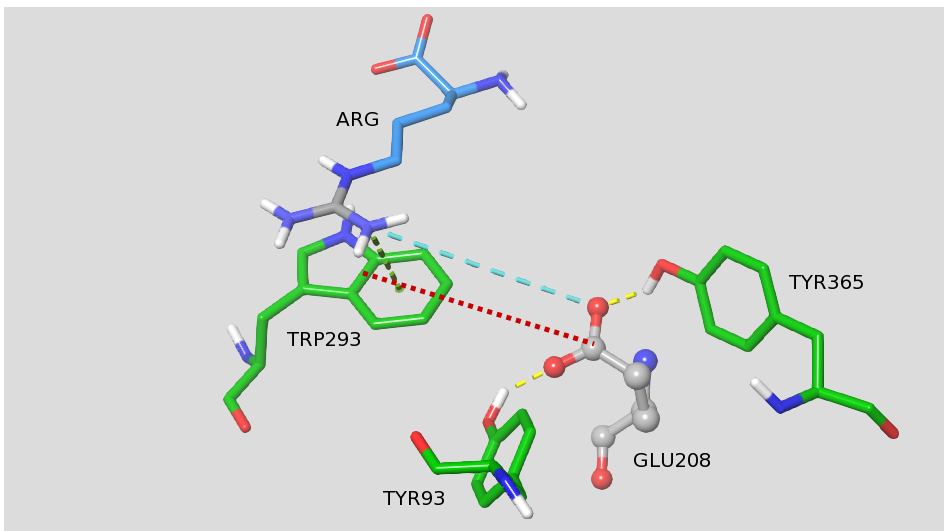


Fig. S2: Interactions formed by Glu208 in the AdiC crystal OF open arginine-bound structure (PDB ID : 3OB6) likely to affect its protonation state. H bonds, ionic medium-range (~8 Å), cation-π and anion-π are depicted as yellow, cyan, green and red dashed lines. All these interactions are also observed in the other substrate-bound structures (PDB IDs: 5J4N, 3L1L) and all but the ionic one are found in the substrate-free structures (PDB IDs: 3LRB, 3NCY, 5J4I).


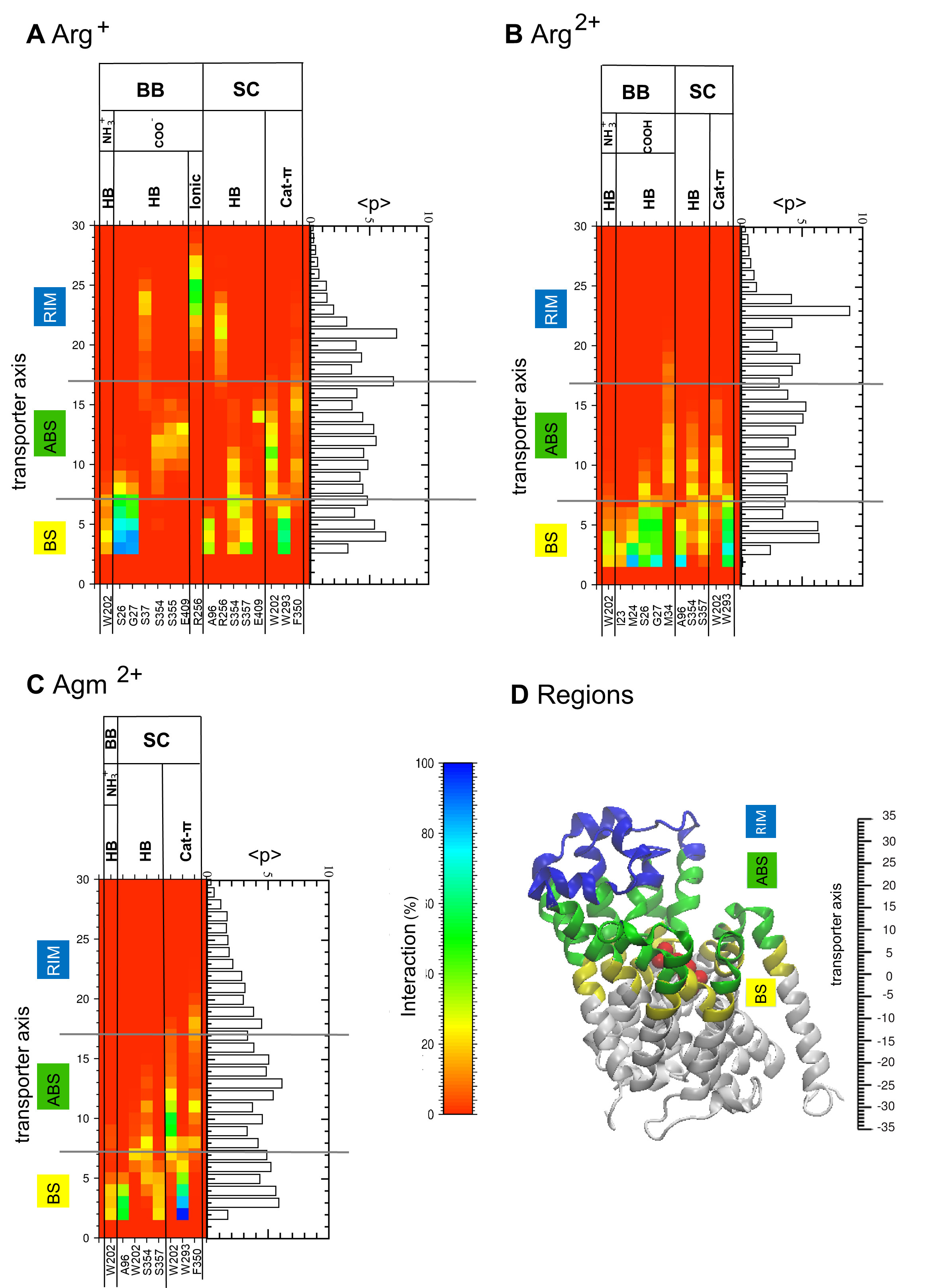


Figure S3: Interactions (H bonds, ionic and cation-π interactions) formed at pH 2:5 (A-C) between the Arg^+^, (A) Arg^2+^ (B), or Agm^2+^ (C) backbone (BB) or sidechain (SC) with protein residues (both BB and SC) during migration of the ligand to the binding site. Also shown is the occurrence of observing the center of mass of the ligand at a certain position along the main axis of the transporter between the external medium and the binding site, as depicted by a bar graph representation. Binding events are depicted for the 18 monomers of the 9 tMD trajectories. Only interactions occurring in more than 20% of the configurations in at least one bin are shown. The abbreviations used for the different interactions are: ionic for ionic interaction, HB for H bond and Cat-π for cation-π interaction. The interaction plots are split into three zones corresponding to (1) the rim of the protein funnel (RIM, from 30 to 17 Å), (2) the region above the binding site (ABS, from 17 to 6 Å), and (3) the binding site (BS, from 6 to 0 Å). (D) The locations of the RIM, ABS, and BS are highlighted on the protein structure in blue, green, and yellow, respectively. The protein structure (PDB ID: 3OB6) is represented as a cartoon and the bound Arg ligand as red spheres. The locations of the different regions along the main axis of the transporter are highlighted by a scale bar.


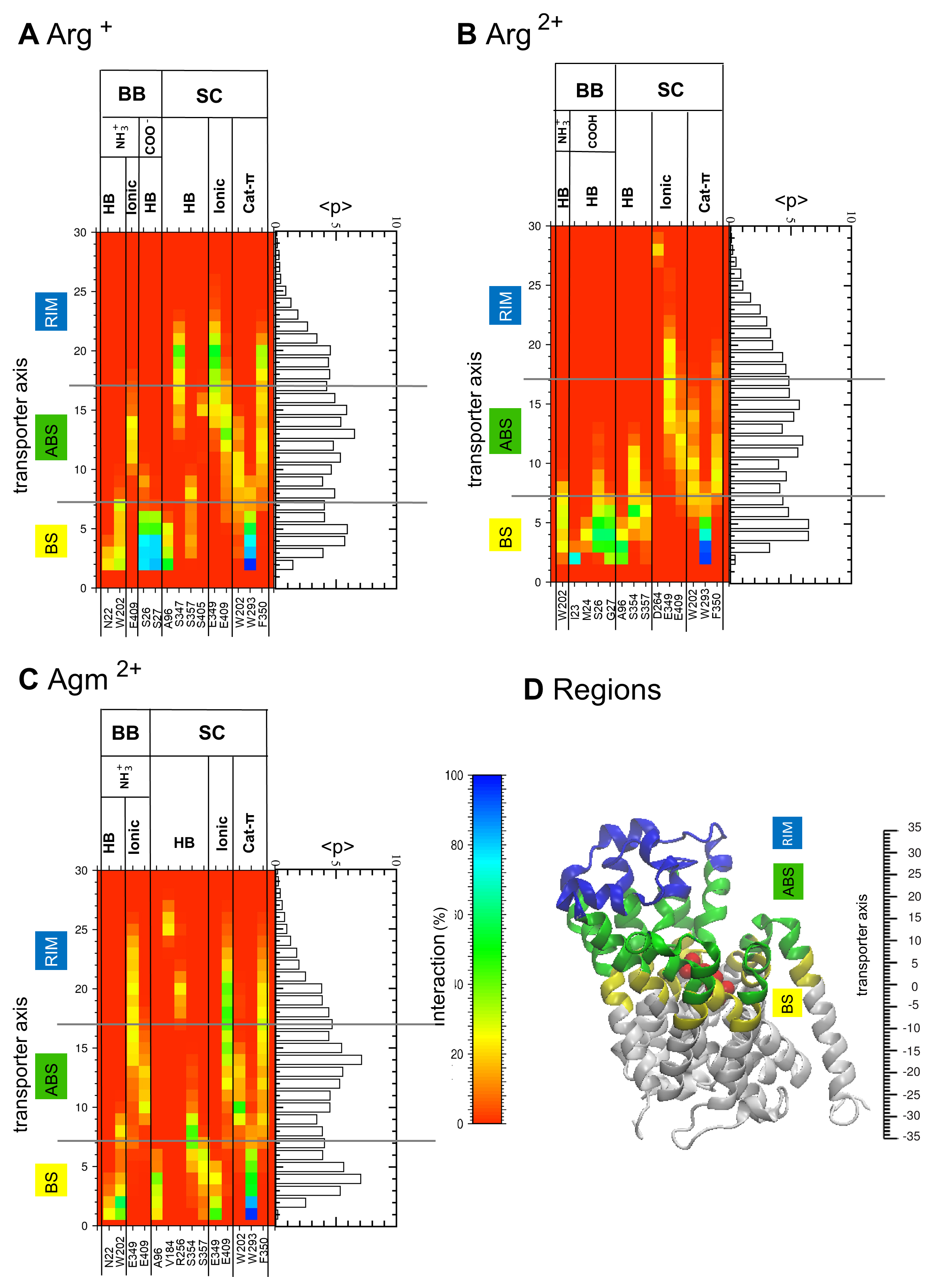


Fig. S4: Interactions (H bonds, ionic and cation-π interactions) formed at pH 6 (A-C) between Arg^+^, (A) Arg^2+^ (B), and Agm^2+^ (C) backbone (BB) and sidechain (SC) and protein residues (both BB and SC) during their migration down to the binding site are shown, along with the occurrence of observing the center of mass of the ligand at a certain position along the main axis of the transporter between the external medium and the binding site, as depicted by a bar graph representation. Only interactions with an occurrence higher than 20% in at least one bin width from all 18 binding events in the 9 tMD trajectories are shown. The abbreviations used for the different interactions are: ionic for ionic interaction, HB for H bond and Cat-π for cation-π interaction. Residues found to interact with Arg in the OF open crystal structure (Monomer A) are highlighted in yellow in (A). The interaction plots are split in three regions (1) the rim of the protein funnel (RIM, from 30 to 17 Å), (2) above the binding site (ABS, from 17 to 6 Å) and in the binding site (BS, from 6 to 0 Å), respectively. (C) The locations of the RIM, ABS and BS are highlighted in blue, green and yellow on the protein structure. The protein structure (PDB ID: 3OB6) is shown as cartoon and the bound Arg ligand as red spheres. The location of the different regions along the main axis of the transporter is highlighted by a scale bar.


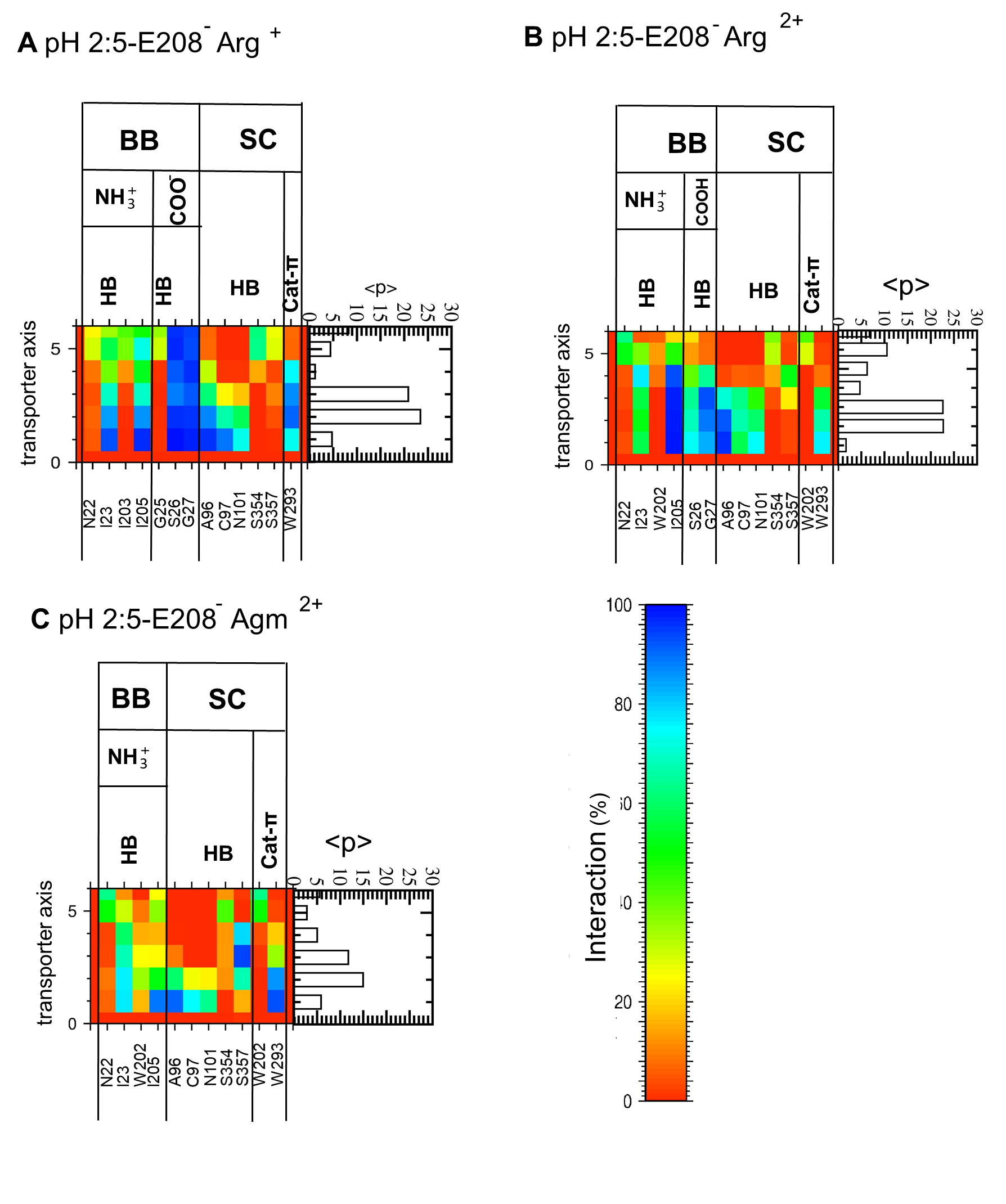


Fig. S5: Interactions (H bonds, ionic and cation-π interactions) formed at pH2:5-E208^-^ (with charged Glu208) between Arg^+^, (A) Arg^2+^ (B), and Agm^2+^ (C) backbone (BB) and sidechain (SC) and protein residues (both BB and SC) in the classical simulations are shown, along with the occurrence of observing the center of mass of the ligand at a certain position along the main axis of the transporter between the external medium and the binding site, as depicted by a bar graph representation. Only interactions with an occurrence higher than 20% in at least one bin width from all 12 monomers of the 6 classical trajectories are shown.

Table S1: Residues predicted to feature different protonation states between the uniform pH 6 and the pH2:5 gradient. A value of 0, 1 and 2, stand for unprotonated, singly and doubly protonated, respectively. All values have been predicted based the OF open structure[2] in which the ligand was removed prior to the calculations.

| **Residue** | **uniform pH 6** | **pH 2/5 gradient** |
| --- | --- | --- |
| Asp121 | 0 | 1 |
| Asp264 | 0 | 1 |
| Asp272 | 0 | 1 |
| Asp306 | 0 | 1 |
| Glu208 | 0 | 1 |
| Glu349 | 0 | 1 |
| Glu409 | 0 | 1 |
| Arg149 | 1 | 1 |
| His8 | 1 | 2 |
| His377 | 1 | 2 |
| His379 | 1 | 2 |
| His432 | 1 | 2 |

Table S2: Interactions formed by arginine with residues in monomer A in the OF open structure [2].

| arginine |  | Interaction type | Residue |
| --- | --- | --- | --- |
| Backbone | COO^-^ | H Bond | S26, G27, M104 |
|  | NH_3_^+^ | H Bond | M24, W202, I205 |
| Sidechain | Gdm^+^ | H Bond | A96, G100, S357 |
|  |  | Cation-π | W293 |

Table S3: Hydration number of each ligand in the binding site region (0<z<6 Å) computed from all classical MD trajectories at pH 2:5, pH 2:5-E208^-^ and pH 6. The hydration number is defined as all water molecules within 4 Å of any ligand heavy atom. The standard errors are also given.

| pH condition | Arg^+^ | Arg^2+^ | Agm^2+^ |
| --- | --- | --- | --- |
| pH2:5 | 12.2±0.02 | 13.2±0.04 | 13.1±0.02 |
| pH2:5-E208^-^ | 10.9±0.18 | 12.1±0.02 | 10.3±0.04 |
| pH6 | 10.2±0.02 | 11.4±0.02 | 11.5±0.04 |

Table S4: Average residence time (in ns) of each ligand in the classical trajectories obtained in different pH conditions calculated as the fraction of conformations in which the center of mass of all ligand heavy atoms is located in the binding site (0<z<6 Å) at pH2:5 or pH2:5-E208^-^. The standard error of the mean is listed in addition.

|  | **Classical MD** | |
| --- | --- | --- |
|  | **pH2:5** | **pH2:5-E208^-^** |
| Arg^+^ | 10.2 ± 2.7 | 11.9 ± 2.6 |
| Arg^2+^ | 6.4 ± 2.3 | 12.8 ± 2.3 |
| Agm^2+^ | 6.0 ± 2.4 | 8.6 ± 2.5 |

Table S5: Average distance (in Å) between Glu208 sidechain and Gmd^+^ of each ligand computed between the center of mass of the two sidechain oxygen atoms of Glu208 and that of the ligand sidechain nitrogen atoms from all classical MD trajectories at pH 2:5, pH 2:5-E208^-^ and pH6 using vmd. The average location of the ligand center of mass in the binding site is also listed. All distances are given in Å. The standard error of the mean is listed in addition.

|  | pH2:5-E208^-^ | | | pH6 | | |
| --- | --- | --- | --- | --- | --- | --- |
|  | Arg^+^ | Arg^2+^ | Agm^2+^ | Arg^+^ | Arg^2+^ | Agm^2+^ |
| Average distance between  Glu208 sidechain and the ligand Gdm^+^ | 8.1±0.01 | 8.9±0.02 | 9.5±0.02 | 8.7±0.02 | 8.1±0.01 | 9.4±0.03 |

Table S6: Contributions to the solvation free energy of protein and complexes with Arg^+^, Agm^2+^ and Arg^2+^ calculated with the QM/IEF-PCM (for monomer A and B separately) and MM-PBSA methods (averaged for monomer A and B) with Glu208 charged (E208^-^) and protonated (E208^0^). All energies are given in kcal/mol.

|  | **QM/IEF-PCM** | | | | | | | **MM-PBSA** | | |
| --- | --- | --- | --- | --- | --- | --- | --- | --- | --- | --- |
|  | **A E208^-^** | | | | **B E208^-^** | | | **A+B E208^-^** | | |
|  | Arg^+1^ | Agm^+2^ | | Arg^+2^ | Arg^+1^ | Agm^+2^ | Arg^+2^ | Arg^+1^ | Agm^+2^ | Arg^+2^ |
| ΔG_solv,complex_ | 116.6 | 103. | | 107.7 | 78.5 | 57.3 | 61.6 | -64.6 | -82.3 | -77.6 |
| ΔG_solv,complex_ polar | -103.7 | -114.8 | | -113.9 | -105.2 | -122.9 | -123 | -79.0 | -96.6 | -94.0 |
| ΔG_solv,complex_ apolar | 220.3 | 217.8 | | 221.6 | 183.7 | 180.2 | 184.5 | 14.4 | 14.3 | 14.4 |
|  |  |  | |  |  |  |  |  |  |  |
| ΔG_solv,p_ | 92.5 | 92.5 | | 92.5 | 59.4 | 59.4 | 59.4 | -67.8 | -66.1 | -65.0 |
| ΔG_solv,p_ polar | -106.8 | -106.8 | | -106.8 | -105.1 | -105.1 | -105.1 | -82.9 | -80.9 | -81.7 |
| ΔG_solv,p_ apolar | 199.3 | 199.3 | | 199.3 | 164.5 | 164.5 | 164.5 | 15.1 | 14.8 | 15.0 |
|  | **A E208^0^** | | | | **B E208^0^** | | | **A+B E208^0^** | | |
|  | Arg^+1^ | | Agm^+2^ | Arg^+2^ | Arg^+1^ | Agm^+2^ | Arg^+2^ | Arg^+1^ | Agm^+2^ | Arg^+2^ |
| ΔG_solv,complex_ | 112.2 | | 72.7 | 79.8 | 73 | 32 | 35.3 | - 65.5 | -111.8 | -105.3 |
| ΔG_solv,complex_ polar | -108.7 | | -143.2 | -141.2 | -110.8 | -148.6 | -149 | -80.1 | -126.7 | -119.9 |
| ΔG_solv,complex_ apolar | 220.8 | | 215.8 | 221.1 | 183.8 | 180.6 | 184.2 | 14.6 | 14.9 | 14.5 |
|  |  | |  |  |  |  |  |  |  |  |
| ΔG_solv,p_ | 112.9 | | 112.9 | 112.9 | 77.9 | 77.9 | 77.9 | -40.5 | -39.9 | -39.6 |
| ΔG_solv,p_ polar | -86.6 | | -86.6 | -86.6 | -87.3 | -87.3 | -87.3 | -55.6 | -55.4 | -54.7 |
| ΔG_solv,p_ apolar | 199.5 | | 199.5 | 199.5 | 165.2 | 165.2 | 165.2 | 15.1 | 15.4 | 15.2 |

Table S7: A. Contributions from changes in configurational (translational, rotational, vibrational) entropy upon ligand binding from RRHO calculated on monomer A only. B. Difference in conformational entropy contribution to the free energy of binding (TΔΔS_conform_) for Arg^2+^ and Agm^2+^, with respect to Arg^+^ used as a reference, calculated on monomer A only. In parenthesis are given the number of rotatable bonds of the divalent versus monovalent ligand. A penalty of 1kcal/mol is applied per bond. C. Difference in binding free energy calculated using a QM procedure and including the entropy contributions for Arg^2+^ and Agm^2+^ with respect to Arg^+^ used as a reference. All values are given in kcal/mol. D. Contributions from changes in configurational entropy upon ligand binding using the quasi harmonic analysis. E. Difference in binding free energy of binding calculated using MM-PBSA and including the vibrational entropy contribution calculated with a quasi-harmonic analysis for Arg^2+^ and Agm^2+^ with respect to Arg^+^ used as a reference. All values are given in kcal/mol.

A.

|  |  | **Arg^+^** | | | **Agm^2+^** | | | **Arg^2+^** | | |
| --- | --- | --- | --- | --- | --- | --- | --- | --- | --- | --- |
| Structure | Glu208  charge | TΔS_trans_ | TΔS_rot_ | TΔS_vib_ | TΔS_trans_ | TΔS_rot_ | TΔS_vib_ | TΔS_trans_ | TΔS_rot_ | TΔS_vib_ |
| A | -1 | -10.6 | -9.6 | 4.8 | -10.3 | -9.1 | 5.2 | -10.6 | -9.6 | 4.9 |
| A | 0 | -10.6 | -9.6 | 4.8 | -10.3 | -9.1 | 5.2 | -10.6 | -9.6 | 4.6 |

B.

|  |  | **Agm^2+^** | **Arg^2+^** |
| --- | --- | --- | --- |
| structure | Glu208 charge | TΔΔS_conform_ | TΔΔS_conform_ |
|  |  | 1 (6/7) | -1 (8/7) |

C.

|  |  | **Agm^2+^** | **Arg^2+^** |
| --- | --- | --- | --- |
| structure | Glu208 charge | ΔΔG_binding_ | ΔΔG_binding_ |
| A | -1 | 12 | 2.9 |
| B | -1 | 8.2 | 8.9 |
| A | 0 | 15.3 | 18.2 |
| B | 0 | 18.8 | 19.1 |

D

|  |  | **Arg^+^** | **Agm^2+^** | **Arg^2+^** |
| --- | --- | --- | --- | --- |
| Structure | Glu208  charge | TΔS_conf_ | TΔS_conf_ | TΔS_conf_ |
| A+B | -1 | 14.5 | 9. | 1.7 |
| A+B | 0 | 25.2 | 17 | -0.6 |

E.

|  |  | **Agm^2+^** | **Arg^2+^** |
| --- | --- | --- | --- |
| structure | Glu208 charge | ΔΔG_binding_ | ΔΔG_binding_ |
| A+B | -1 | 67.3 | 52.3 |
| A+B | 0 | 89.4 | 84.2 |

|  |  | **Arg^+^** | | | |
| --- | --- | --- | --- | --- | --- |
| Structure | Glu208 charge | $\Delta E_{\mathrm{int}}$ | ΔG_solv,ligand_ | ΔG_solv,p_  polar | ΔG_solv,complex_ polar |
| A+B | -1 | -134.2 ± 0.7 | -102.6 ± 0.2 | -82.9 ± 0.1 | -79.0 ± 0.2 |
| A+B | 0 | -85.6 ± 0.9 | -97.2 ± 0.2 | -55.6 ± 0.0 | -80.1 ± 0.2 |
|  |  | **Agm^2+^** | | | |
| Structure | Glu208 charge | $\Delta E_{\mathrm{int}}$ | ΔG_solv,ligand_ | ΔG_solv,p_  Polar | ΔG_solv,complex_ polar |
| A+B | -1 | -134.6 ± 0.9 | -187.4 ± 0.1 | -80.9 ± 0.1 | -96.6 ± 0.3 |
| A+B | 0 | -47.0 ± 0.9 | -186.6 ± 0.1 | -55.4 ± 0.1 | -126.7 ±0.4 |
|  |  | **Arg^2+^** | | | |
| Structure | Glu208 charge | $\Delta E_{\mathrm{int}}$ | ΔG_solv,ligand_ | ΔG_solv,p_  Polar | ΔG_solv,complex_ polar |
| A+B | -1 | -162.4 ± 0.8 | -186.4 ± 0.2 | −81.7 ± 0.1 | -94.0 ± 0.4 |
| A+B | 0 | -75.0 ± 1.1 | -185.7 ± 0.1 | -54.7 ± 0.0 | -119.9 ± 0.3 |

Table S8: Free energies of binding calculated using MM-PBSA. (A) ΔE_int_, ΔG_solv,complex_, ΔG_solv,p,_ and ΔG_solv,ligand_ for Arg^+^, Arg^2+^, and Agm^2+^ and their standard errors. All energies were averaged for the two monomers (A and B) and computed for both Glu208 protonation states. All energy values are given in kcal/mol.

Table S9: List of the titratable residues and their connectivity to either the periplasm (P) or cytoplasm (C) used in the protonation probability calculations in a pH gradient.

| Asp6 | C | His8 | C |
| --- | --- | --- | --- |
| Lys9 | C | Tyr44 | P |
| Tyr60 | C | Lys62 | C |
| Asp67 | C | Tyr74 | C |
| Tyr76 | C | Arg78 | C |
| Arg79 | C | Cys80 | C |
| Tyr87 | C | Tyr93 | P |
| Cys97 | P | Tyr111 | P |
| Tyr144 | P | Lys120 | P |
| Asp121 | P | Cys129 | P |
| Lys145 | C | Arg149 | C |
| Arg174 | P | Glu176 | P |
| Tyr178 | P | Glu208 | P |
| Lys218 | C | Lys221 | C |
| Arg222 | C | Cys238 | P |
| Tyr239 | P | Arg256 | P |
| Asp264 | P | Arg267 | P |
| Asp272 | P | Cys281 | P |
| Cys286 | P | Lys302 | C |
| Asp306 | C | Asp307 | C |
| Arg316 | C | Lys319 | C |
| Lys348 | P | Glu349 | P |
| Tyr365 | P | Tyr367 | C |
| Cys369 | C | His377 | C |
| His379 | C | Lys282 | C |
| Arg384 | C | Tyr387 | C |
| Tyr397 | C | Lys382 | C |
| Arg384 | C | Tyr387 | C |
| Tyr397 | C | Cys398 | C |
| Lys408 | P | Glu409 | P |
| Tyr424 | C | Tyr428 | C |
| Arg430 | C | His432 | C |
| Lys433 | C | Tyr436 | C |
| Asp439 | C |  |  |
